# Genome wide mapping of *ETV6* binding sites in pre-B leukemic cells

**DOI:** 10.1038/s41598-018-33947-1

**Published:** 2018-10-19

**Authors:** Benjamin Neveu, Maxime Caron, Karine Lagacé, Chantal Richer, Daniel Sinnett

**Affiliations:** 1 Sainte-Justine UHC Research Center, Montreal, Qc Canada; 2 Department of Biochemistry and Molecular Medicine, Faculty of Medicine, University of Montreal, Montreal, Qc Canada; 3 Department of Pediatrics, Faculty of Medicine, University of Montreal, Montreal, Qc Canada

## Abstract

Genetic alterations in the transcriptional repressor *ETV6* are associated with hematological malignancies. Notably, the t(12;21) translocation leading to an *ETV6*-AML1 fusion gene is the most common genetic alteration found in childhood acute lymphoblastic leukemia. Moreover, most of these patients also lack *ETV6* expression, suggesting a tumor suppressor function. To gain insights on *ETV6* DNA-binding specificity and genome wide transcriptional regulation capacities, we performed chromatin immunoprecipitation experiments coupled to deep sequencing in a t(12;21)-positive pre-B leukemic cell line. This strategy led to the identification of *ETV6*-bound regions that were further associated to gene expression. *ETV6* binding is mostly cell type-specific as only few regions are shared with other blood cell subtypes. Peaks localization and motif enrichment analyses revealed that this unique binding profile could be associated with the *ETV6*-AML1 fusion protein specific to the t(12;21) background. This study underscores the complexity of *ETV6* binding and uncovers *ETV6* transcriptional network in pre-B leukemia cells bearing the recurrent t(12;21) translocation.

## Introduction

*ETV6* is a member of the ETS superfamily of transcription factors that are critical modulators of cellular homeostasis in several tissues. The normal function of ETS factors is mandatory for appropriate cell fate as dysregulation or deleterious events affecting these factors are frequently observed in a variety of cancers^1^. *ETV6* is essential in the establishment and maintenance of hematopoiesis within the bone marrow compartment^2,3^. *ETV6* translocations are frequently observed in various hematological disorders^4^ and germline mutations have been associated to predispositions for such diseases^5–10^.

The most common *ETV6* aberration is the t(12;21)(p13;q22) translocation which fuses *ETV6* to the *AML1* gene (or *RUNX1*) and generates an in-frame *ETV6*-*AML1* chimeric protein^11^. This is the most frequent chromosomal abnormality in childhood pre-B cell acute lymphoblastic leukemia (pre-B ALL), occurring in 20% of cases^12^. However, the *ETV6*-*AML1* fusion protein seems insufficient to induce leukemia by itself^13–15^, suggesting that additional events are required to fully develop pre-B ALL^16^. Interestingly, the complete inactivation of *ETV6* in t(12;21)-positive pre-B ALL cases was underscored by several studies^17–21^ and indicates that *ETV6* depletion could lead to pre-B ALL initiation.

Unlike the majority of ETS members, *ETV6* acts as a transcriptional repressor^22,23^. *ETV6* has a N-terminal pointed (PNT) helix-loop-helix domain required for protein-protein interactions and homodimerization^24^. Its central repressive domain is also implicated in protein-protein interactions with members of the SMRT/N-CoR/mSin3A/HDAC co-repressor complexes^25–27^. The C-terminal part of *ETV6* contains an ETS DNA-binding domain that recognizes a consensus ETS-binding site consisting of a core GGAA/T sequence with adjacent purine-rich sequences^24^. Interestingly, the *ETV6*-*AML1* fusion protein combines both PNT and central repressive domains of *ETV6* with Runt DNA binding and transactivation domains of *AML1*, thus converting *AML1* from a transcriptional activator to a putative repressor^28^.

Although the molecular functions of *ETV6*-*AML1* have been studied^29–31^, the exact role of *ETV6* remains poorly understood. To gain insights into *ETV6* function in t(12;21)-positive pre-B leukemia cells, we sought to identify *ETV6* binding sites using chromatin immunoprecipitation coupled to high throughput sequencing (ChIP-seq). By including expression data^32^, we extensively described *ETV6* binding properties and transcriptional activity in this particular context.

## Methods

### Constructs

The complete wild-type coding sequence of *ETV6* was subcloned into pcDNA3.1 (pcDNA3.1 *ETV6*). The C-terminal HA-tagged *ETV6* construct was generated by restriction enzyme digestion as described previously^32^ using an oligomer containing 3 tandem HA tag repeats. Both *ETV6* and *ETV6*-HA were subcloned into pCCL lentiviral vector (kindly provided by Dr. Christian Beauséjour) through enzymatic digestion and ligation.

### Cell culture

Reh (ATCC ® CRL-8286™), a t(12;21)-positive pre-B ALL cell line, was maintained in RPMI 1640 (Wisent) 10% Fetal Bovine Serum (FBS; Wisent) in a 5% CO_2_ incubator at 37 °C.

### Lentiviral production

1.5 × 10^7^ HEK293T cells were seeded into 15 cm petri dishes in DMEM (Wisent) 10% FBS. The next day, cells were transfected with 9 μg pCCL plasmids together with 6 μg pRSV-Rev, 7.8 μg pMD2.VSVG and 15 μg pMDL third generation encapsidation plasmids (kindly provided by Dr. Christian Beauséjour) in fresh RPMI 1640 10% FBS medium using polyethylenimine (Polysciences) at a final concentration of 6.5 μg/mL. Media was removed 16 h post-transfection and replaced by fresh DMEM 10% FBS. After 30 h, viral particles were retrieved from media by ultracentrifugation (50 000 g) and quantified by p24 antigen ELISA (Advanced Bioscience Laboratories).

### Lentiviral infection

2 × 10^6^ Reh cells and two different Reh clones (generated in methylcellulose media) were seeded in 2 mL of RPMI 1640 10% FBS medium. 200 ng of concentrated virus were added to cells with polybrene (Sigma) to a final concentration of 8 μg/mL. 24 h post-infection, medium was changed with fresh RPMI 1640 10% FBS. These cells were maintained 2 weeks in culture before carrying out further experiments.

### Western blotting

20 μg of nuclear protein extracts were diluted in Laemmli buffer and migrated on SDS-denaturating 10% polyacrylamide gels. Transfer on polyvinylidene difluoride membranes was performed at 4 °C overnight. Membranes were blocked in Blotto A solution (1X TBS, 5% milk and 0.05% Tween-20) prior to immunoblotting using the primary antibodies against *ETV6* (1:1000; ab54705; Abcam) or GAPDH (1:1000; sc-31915; Santa Cruz) and HRP-coupled secondary antibodies anti- mouse (1:5000; sc-358914; Santa Cruz) and anti-goat (1:5000; sc-2961; Santa Cruz) IgG, respectively. Membranes were then assayed by enhanced chemiluminescence detection with Western Lightning Plus-ECL (PerkinElmer) according to the manufacturer protocol.

### Chromatin immunoprecipitation

Chromatin immunoprecipitation (ChIP) has been performed as previously described^32^. Briefly, cross-linked chromatin isolated from 1.0 × 10^7^ transduced Reh cells was used for immunoprecipitation with anti-HA magnetic beads (Thermo Fisher Scientific). DNA-protein complexes were eluted from the beads by competition with HA peptides prior to reverse-crosslinking and standard purification using phenol/chloroform/isoamyl alcohol (Sigma). The purified ChIP DNA was processed through TruSeq ChIP Sample Preparation Kit (Illumina) according to the manufacturer protocol. As positive control, a fraction of the amplified ChIP material was used to assess the *ETV6* binding enrichment at the *CLIC5A* promoter^32^ by quantitative PCR (qPCR) using primers listed in Supplementary Table S1. Libraries were sequenced on the HiSeq 2500 system (Illumina) in paired-end mode (2 × 100 bp).

### ChIP-seq data analysis

Raw reads were aligned on the Hg19 reference genome using bowtie v2.2.3^33^ and filtered using a mapping quality threshold of 20. Reads were merged for both *ETV6* and *ETV6*-HA populations (n = 3). Peaks were called in the *ETV6*-HA condition over the background obtained in the *ETV6* condition (negative IP control) using MACS v2.1.1.20160309^34^ with a q-value threshold of 0.1. Peaks overlapping blacklisted regions (ENCODE’s EncodeDacMapabilityConsensus^35^, svelter’s exclude file^36^ and canva’s filter file^37^) or having a fold enrichment above 50 were discarded. Peaks were associated to ensembl genes using the closest gene transcription start site (TSS) annotations from HOMER and genomic coordinates from bedops’ closest-features v2.4.19^38^. To define the minimal peak fold enrichment threshold (≥4.5), a Fisher’s exact test *p-value* was calculated for the overlaps between *ETV6* peaks and *ETV6*-modulated genes^32^ against the proportion of *ETV6* peaks in the complete ensembl genes repertoire. Genomic annotations of peaks and motif enrichment analyses were performed using HOMER v4.8^39^.

*ETV6* ChIP-seq data in GM12878 and K562 cells are publically available as part of the ENCODE project.

GM12878:https://www.encodeproject.org/files/ENCFF272DJU/@@download/ENCFF272DJU.bed.gz

K562:https://www.encodeproject.org/files/ENCFF514SLV/@@download/ENCFF514SLV.bed.gz

*ETV6* peaks in GM12878 and K562 cells were thus obtained directly from the ENCODE platform as conservative IDR bed files (narrowPeak). The intersection of *ETV6* peaks between cell types was obtained using pybedtools v.0.7.7 venn_mpl with default parameters^40^.

Chromatin state data of GM12878 cells were obtained from the NIH Roadmap Epigenomics Project Portal (E116, 15 coreMarks)^41^.

## Results

### Identification of genome wide *ETV6*-bound regions

**Figure 1:**
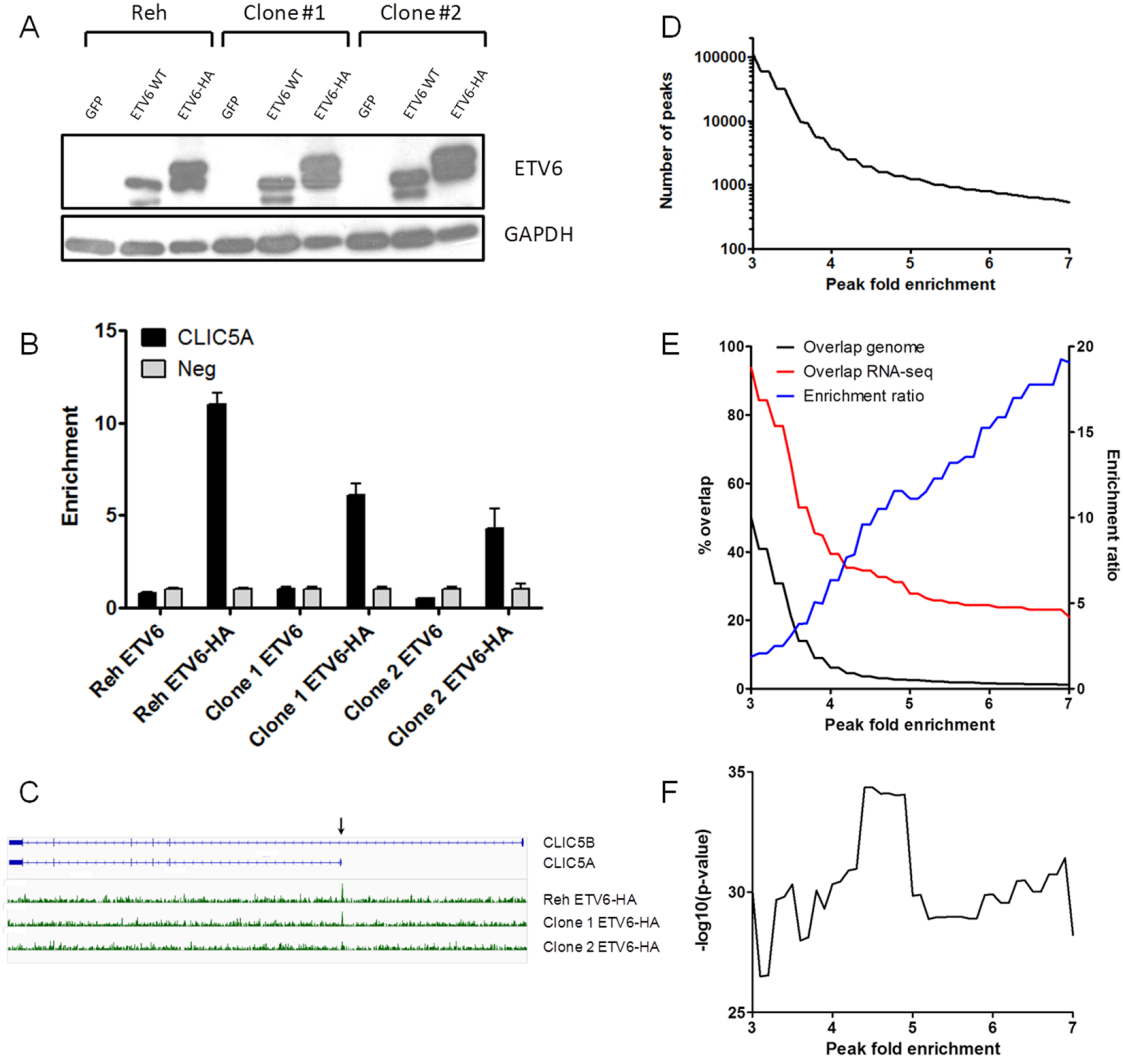
ChIP-seq analysis of *ETV6* binding sites in pre-B leukemia cells. (**A**) The Reh cell line, a pre-B leukemia cell line which is t(12;21)-positive and *ETV6* negative, was transduced with either pCCL-GFP, *ETV6* or *ETV6*-HA. The expression of *ETV6* and *ETV6*-HA was confirmed by western blot. Results from two derived clones are shown. *This blot was cropped and* a*djusted for brightness and contrast. The original blot is shown in the supplementary information*. (This figure is the same control Western Blot as the one we published as Supplementary Figure S2 in Haematologica. 2016 Dec;101(12):1534 – 1543 PMID:27540136^32^; with permission of the Ferrata Storti Foundation©. The exact same cells were used in both publications). (**B**) ChIP-qPCR analysis of the *CLIC5* locus from the HA-immunoprecipitated DNA. The *CLIC5A* promoter, a known bound region of *ETV6*^32^, was successfully enriched in *ETV6*-HA populations compared to a negative control region (neg), but not in the untagged *ETV6* populations. Error bars represent the standard deviation (n = 4) (**C**). Distribution of the reads mapped to the *CLIC5* gene after sequencing of the HA-immunoprecipitated DNA. A ChIP signal at the *CLIC5A* promoter region (arrow) is seen in all three *ETV6*-HA populations. (**D**) The number of *ETV6* ChIP peak signals using variable peak fold enrichment thresholds (0.1 increments in a 3 to 7 range). The number of peaks drastically increases with peak fold enrichment thresholds <4. (**E**) Overlap percentage (left *y* axis) between the genes associated with putative *ETV6* binding sites and known *ETV6*-modulated genes^32^ (red line) or total ensembl genes (black line). The ratio between the percentages of overlap was calculated (blue line; right *y* axis) and increases with the stringency of the peak fold enrichment thresholds. (**F**) A Fisher’s exact test *p-value* was calculated for the overlaps between *ETV6* peaks and *ETV6*-modulated genes against the proportion of *ETV6* peaks in the complete ensembl genes repertoire. The most significant enrichment is calculated at a peak fold enrichment of ≥4.5.

To determine the genomic regions bound by *ETV6*, we expressed *ETV6* and HA-tagged *ETV6* in 3 biological replicates of Reh pre-B leukemic cells (Fig. 1A). Of note, Reh cells lack endogenous wild-type *ETV6* expression as a result of a t(12;21) translocation and a 12p13 locus deletion. These cells were then used for ChIP experiments using the HA epitope as a bait. We confirmed *ETV6* binding to the *CLIC5A* promoter region, a validated *ETV6* target gene^32^, in the 3 replicates, both by qRT-PCR (Fig. 1B) and high throughput sequencing (Fig. 1C), indicating that our ChIP-seq data are suitable for the identification of *ETV6*-bound regions. Note that the mapping and the peak calling procedures were performed individually for each sample to confirm the reproducibility of the biological replicates (Supplementary Fig S1). To retrieve the maximum of significant and robust *ETV6* peaks, the reads from the replicates were pooled.

The number of *ETV6* peaks called varies markedly with the fold enrichment threshold (Fig. 1D; Supplementary Table S2). Effective *ETV6* binding is expected to impact the expression of nearby genes. Accordingly, *ETV6* peaks were associated to genes and compared to known *ETV6*-modulated genes (88 downregulated and 59 upregulated genes following *ETV6*-His expression in Reh cells) obtained by RNA-seq^32^. This strategy was performed through the entire peak fold enrichment range (Fig. 1E; Supplementary Table S2). The overlap between *ETV6* peaks and *ETV6*-modulated genes is systematically higher than the random probability given by the proportion of *ETV6* peaks in the complete ensembl genes repertoire. The ratio calculated between these overlaps is directly correlated to the peak fold enrichment (Fig. 1E, enrichment ratio; Supplementary Table S2), indicating that stronger *ETV6* peaks are more likely associated with *ETV6*-mediated transcriptional regulation.

A peak fold enrichment ≥4.5 shows the strongest peak-to-gene expression association (Fisher’s exact test *p-value* = 4.46E^−35^; Fig. 1F; Supplementary Table S2). Using 4.5 as a specified threshold, 1,931 peaks were associated to 2,223 genes (Supplementary Table S3), representing 3.6% of the total ensembl genes set (n = 60,235). However, 51 of the 147 (34.6%) known *ETV6*-modulated genes in Reh cells^32^ have at least one associated peak called in this condition (74 expression-correlated peaks; Supplementary Table S4). By including expression data, we thus selected 1,931 high priority *ETV6*-bound regions that were used in the subsequent analyses.

### *ETV6* distribution across hematopoietic cell lines genomes

**Figure 2:**
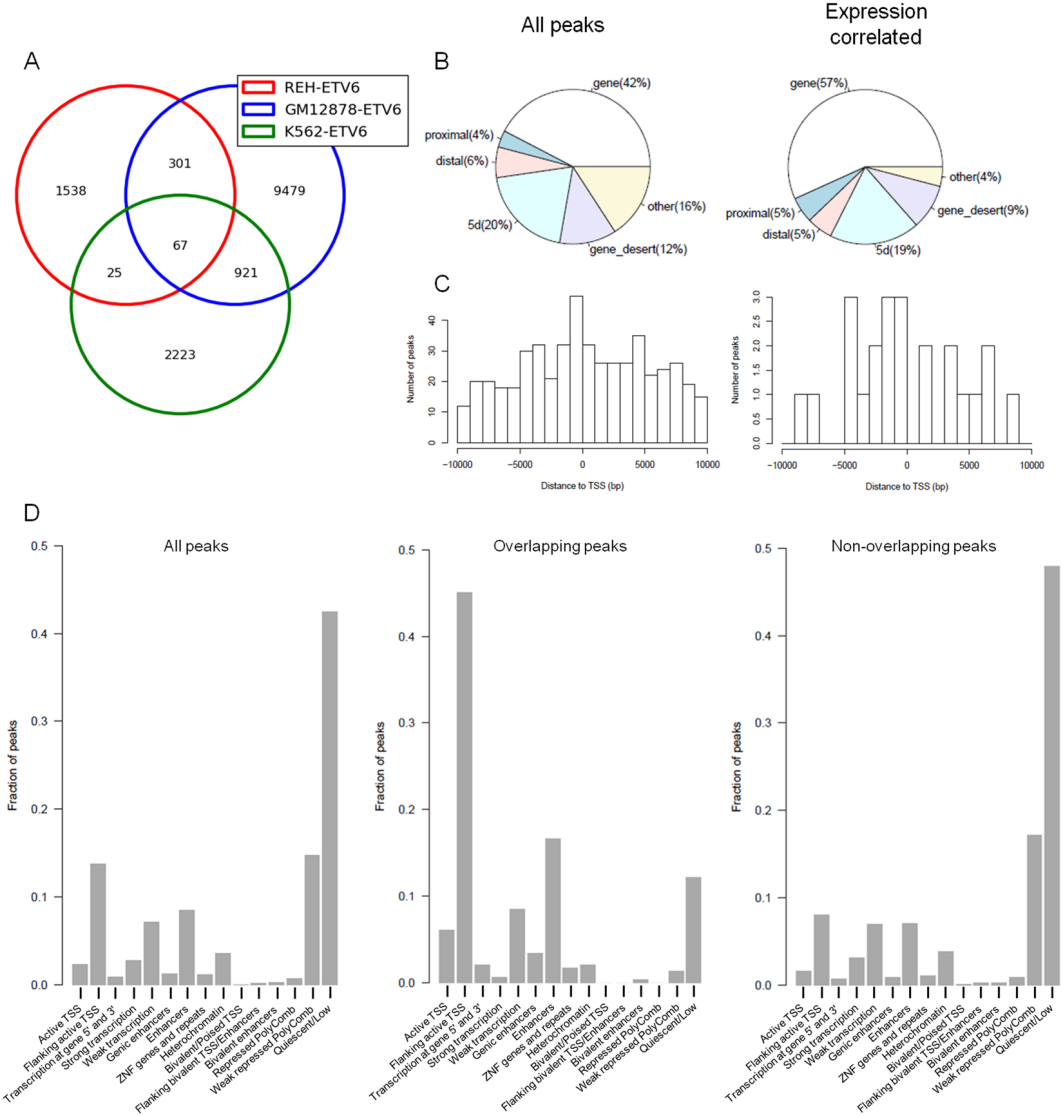
Genomic annotations of *ETV6*-bound regions. (**A**) Comparison of *ETV6* binding sites in Reh-*ETV6* cells and two other hematopoietic cell lines, GM12878 and K562. (**B**) Genomic distribution of all Reh-derived *ETV6* peaks (left panel; n = 1,931) and expression-correlated peaks (right panel; n = 74). Proximal: ≤2 kb upstream of TSS; Distal: 2 kb to 10 kb upstream of TSS; 5d: 10 kb to 100 kb upstream of TSS. *ETV6* binding occurs mostly in genes. (**C)** Distribution of all Reh-derived *ETV6* peaks (left panel) and expression-correlated peaks (right panel) in a 20 kb region across the TSS (+/−10 kb). Expression-correlated peaks are not enriched for proximal TSS binding. (**D**) Left panel: Reh-derived *ETV6*-bound regions were associated to GM12878 chromatin states (*x* axis) based on epigenetic profiles. Center panel: The same analysis was restricted to Reh and GM12878 overlapping *ETV6* peaks. Those shared *ETV6*-bound regions are mainly flanking active TSS regions and enhancers. Right panel: Reh-specific *ETV6* peaks are mostly associated to quiescent regions in GM12878 cells.

We next compared *ETV6*-bound regions identified in pre-B leukemia cells (Reh) to those of normal lymphoblastoid cells (GM12878) and myelogenous leukemia cells (K562) obtained from the ENCODE project (Fig. 2A). The overlap between Reh and GM12878 cells (368 of the 1,931 Reh peaks; 19.05%) was greater than with K562 cells (92 of the 1,931 Reh peaks; 4.76%), suggesting that the lineage (lymphoid vs. myeloid) is more likely to shape *ETV6* binding compared to the differentiation stage (pre-B vs. B). The strongest overlap was however observed between K562 and GM12878 cells (988 of the 3,236 K562 peaks; 30.53%). Of note, the same antibody and immunoprecipitation protocol were used to generate these ENCODE datasets and might favor this overlap. Only 67 regions were bound by *ETV6* in all 3 cell lines (3.47% of Reh peaks). Peaks shared across Reh and GM12878 cells were however not more associated to *ETV6*-modulated genes in Reh^32^ (Supplementary Table S5).

### Genomic features of *ETV6*-bound regions

We next interrogated the genomic localization of *ETV6* peaks. Figure 2B (“All peaks”; left panel) shows the percentage of peaks located within distinct genomic regions. The peaks are more often found (42%) within genes bodies (exons and introns). This distribution was similar in both GM12878 and K562 cells (Supplementary Fig S2). When we assessed the localization of expression-correlated *ETV6* peaks, a stronger association (57%) was observed within gene (Fig. 2B, right panel). *ETV6* binding within genes seems to induce a change in gene expression.

The number of peaks located in the vicinity of the transcription start site (TSS) was assessed (Fig. 2C). Of the 1,931 *ETV6* peaks, 502 (26%) were in the flanking region (+/− 10 kb) of a TSS. The highest number of peaks within this range is observed in the first 1,000 bp upstream of the TSS (Fig. 2C, left panel). The distribution of *ETV6* peaks observed in Reh cells is less TSS centered compared to GM12878 and K562 cells (Supplementary Fig S2). Although the relatively small sample size of expression-correlated *ETV6* peaks, they did not display a tighter distribution around the TSS. (Fig. 2C, right panel), suggesting that *ETV6*-mediated transcription can be induced by distant TSS binding in Reh cells.

Epigenetic modifications in GM12878 cells were extensively investigated^41^ compared to Reh. Considering the number of shared *ETV6* peaks between Reh and GM12878 cell lines (368 peaks, Fig. 2A), we assigned Reh-derived *ETV6* peaks to specific chromatin states based on epigenetic data from the corresponding regions in GM12878 cells. Most of *ETV6* peaks were associated to a quiescent state (Fig. 2D, left panel). However, when focusing on shared Reh and GM12878 *ETV6* peaks, 45% of these were categorized as flanking active TSS (Fig. 2D, center panel; enriched for H3K4me3 and H3K4me1). Enhancers were also significantly enriched (Fig. 2D, center panel; enriched for H3K4me1 and high CpG methylation). In contrast, Reh-specific peaks are depleted for both flanking active TSS and enhancers chromatin states and are instead further associated with quiescent regions in GM12878 cells (Fig. 2D, right panel), indicating that *ETV6* binding to a given region appears to depend on a prerequisite chromatin environment.

### Motifs enrichment of *ETV6*-bound regions

**Figure 3:**
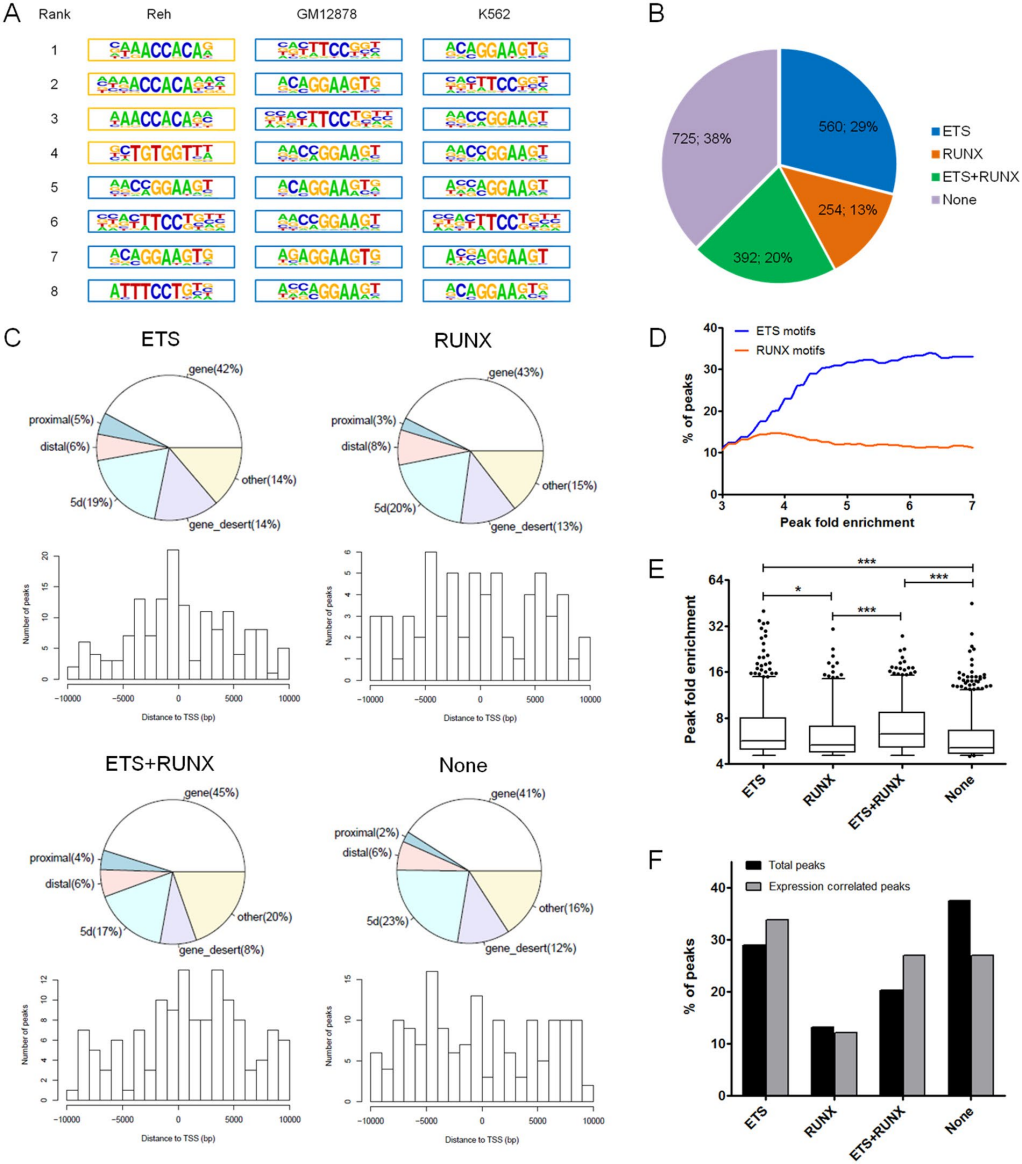
Consensus binding motifs enrichment analyses. (**A**) Consensus binding motifs associated with *ETV6*-bound regions are shown for Reh, GM12878 and K562 cell lines. ETS motifs (blue border) were significantly enriched in all three cell lines. RUNX motifs (orange border) were also significantly enriched in Reh cells. (**B**) Reh-derived *ETV6*-bound regions were classified based on the presence or absence of ETS or RUNX motifs (identified in (**A**) (**C**) Genomic annotations of peaks for each motif groups. Proximal: ≤2 kb upstream of TSS; Distal: 2 kb to 10 kb upstream of TSS; 5d: 10 kb to 100 kb upstream of TSS. Notably, ETS-unique peaks shows a tighter distribution across the TSS region. (**D**) Percentages of *ETV6*-bound regions containing either ETS or RUNX using variable peak fold enrichment thresholds. ETS but not RUNX motifs become increasingly more frequent with stronger peak fold enrichments. (**E**) Fold enrichment of all *ETV6* peaks in Reh cells according to each motif groups. ETS containing peaks (ETS and the ETS/RUNX compound) have significantly greater fold enrichments. Whiskers represent the 5–95 percentiles. Statistical significance is calculated by two-tailed Student’s *t* test. (**F**) Proportion of each motif groups in expression-correlated *ETV6* peaks (n = 74) compared to all *ETV6* peaks (n = 1,931). ETS containing peaks (ETS and the ETS/RUNX compound) are even more over-represented in expression-correlated peaks.

ETS transcription factors such as *ETV6* have well-known binding motifs^24^. As expected, motif enrichment analyses revealed significant over-representation of ETS motifs within *ETV6* peaks in Reh cells as well as in GM12878 and K562 cells (Fig. 3A,B; Supplementary Table S6). Interestingly, based on the ranking of the enriched motifs, RUNX motifs were significantly more over-represented in Reh cells compared to GM12878 and K562 cells (Fig. 3A,B; Supplementary Table S6). RUNX motifs are found centered on the peak summits similarly to ETS motifs (Supplementary Fig S3), indicating that RUNX-containing peaks are also properly bound. Unlike GM12878 and K562 cells, Reh cells express the *ETV6*-*AML1* fusion protein that can interact with the bait protein *ETV6*-HA through their PNT domain and bind RUNX motifs^42^. Therefore, *ETV6* ChIP-seq in this particular t(12;21)-positive background may have retrieved putative *ETV6*-*AML1*-bound regions in addition to *ETV6*-bound regions.

Only minor differences were observed when we assessed the global genomic localization pattern of *ETV6* peaks according to the motif groups (refer to Fig. 3B) in Reh cells (Fig. 3C). However, *ETV6* binding occurs predominantly in the first 1,000 bp upstream of TSS only in ETS-unique peaks (Fig. 3C). With ETS motifs peaks being directly bound by *ETV6*-HA, the intensity (or peak fold enrichment) of these peaks is expected to be higher than RUNX motifs peaks which are presumably bound through *ETV6*-*AML1*. A striking difference is indeed observed when we compared the percentage of peaks containing only ETS (direct binding) or RUNX (indirect binding) motifs according to different peak fold enrichment (Fig. 3D). ETS motifs become increasingly predominant among *ETV6*-bound regions with higher peak fold enrichment thresholds. Inversely, the fraction of *ETV6*-bound regions containing RUNX-unique motifs remains constant through the peak fold enrichment range.

*ETV6* peaks containing ETS motifs (unique or with RUNX) are therefore significantly stronger (i.e. higher fold enrichment) than RUNX-unique peaks and ETS/RUNX depleted peaks (Fig. 3E). Interestingly, peaks correlated with gene expression modulation have significantly higher fold enrichments (Supplementary Table S7) without significant differences between motif groups (Supplementary Fig S4). We then assessed the relationship between motifs and expression. As shown in Fig. 3F, expression-correlated peaks are slightly more enriched for ETS motifs (unique or with RUNX) compared to all the peaks. Inversely, peaks lacking both ETS and RUNX motifs are less likely to induce a change in gene expression. All together, these data indicate that *ETV6* predominantly binds ETS-containing sequences *in vivo*. ETS-driven binding of *ETV6* is generally stronger than other binding events and are even more associated with a change in gene expression.

Although most *ETV6* binding sites contain either ETS or RUNX motifs, a non-negligible fraction does not (38%, Fig. 3B). Additional motif enrichment analyses revealed that only 10 motifs were significantly over-represented among this subset of ETS/RUNX free peaks (q-value < 0.1; Supplementary Table S8). IRF consensus sequences were present in 3 of these enriched motifs with one of them in conjunction with an ETS motif (Supplementary Table S8, see motif #4). Interestingly, this ETS-IRF compound motif was also strongly associated to expression regulation (Supplementary Fig S5). In fact, 10 of the 20 expression-correlated peaks lacking ETS or RUNX motifs instead contain this ETS-IRF compound motif. It is known that *ETV6* can interact with *IRF8*^43^, suggesting a potential regulation through this interaction similarly to what was observed with RUNX motifs and *ETV6*-*AML1*. These results suggest a role for *ETV6* as a cofactor at these loci.

## Discussion

In this study, we provided the first in-depth mapping of *ETV6* binding sites in t(12;21)-positive pre-B leukemia cells. We further associated *ETV6* binding to transcriptional regulation by integrating expression data^32^. Despite the clear correlation between *ETV6* binding and gene expression in the Reh cell line, *ETV6* binding profile in these cells was markedly different of those obtained in other hematopoietic cell types. By including epigenetic data, we were able to unveil the importance of the cell type-specific chromatin environment on *ETV6* binding. In this regard, roughly half of Reh-specific *ETV6* peaks maps in quiescent regions of the lymphoblastoid cell line GM12878. The cell type-specific transcriptional programs and related active chromatin states thus modulate *ETV6* binding. Although ubiquitously expressed, *ETV6* impact on expression profiles might be different across tissues and cell types as its binding depends on higher order chromatin conformations. It is noteworthy that *ETV6* silencing or overexpression in purified CD34^+^ cells from cord blood led to differentially expressed genes with however no recurrence with genes obtained in Reh cells (unpublished observations^32^;), further demonstrating the cell-specific role of *ETV6* in transcriptional regulation.

*ETV6* binding might as well rely on other proteins^43,44^ whose availability would lead to additional complexity and divergences across cell types. For instance, *ETV6*-*AML1* seems to shape *ETV6* binding profile in Reh cells. Although additional experiments could further strengthen our observations, the over-representation of RUNX motifs is likely a consequence of their interaction^42^ as these sequences are expectedly targeted by the Runt domain of the fusion protein^29^. The contrasting distribution across TSS of *ETV6* peaks in Reh cells compared to other cell types may be attributed to this interaction as the dissection per motif groups revealed a tighter distribution of ETS-unique peaks. Furthermore, not only *ETV6* is recruited to RUNX-containing sequences but also induced changes in gene expression at these loci. RUNX-unique motifs were found in similar proportions within *ETV6* peaks and expression-correlated peaks. More importantly, *ETV6* peaks containing both ETS and RUNX consensus binding sequences were even more associated with differentially expressed genes. These regions may be bound synergistically by the complex formed by *ETV6*-HA and endogenous *ETV6*-*AML1* through their ETS and Runt domains, respectively. Interestingly, the expression of *ETV6*^32^ or the knock-down of *ETV6*-*AML1*^31^ in Reh cells lead to common differentially expressed genes (37 of the 147 *ETV6*-modulated genes are also differentially expressed upon *ETV6*-*AML1* knock-down), further suggesting that *ETV6* and *ETV6*-*AML1* cooperate in transcriptional regulation. Additionally, it remains possible that a fraction of RUNX-containing peaks could have been retrieved through the recruitment of *ETV6* to *AML1* itself as several reports described an interaction between *AML1* and other ETS transcription factors^45–48^.

In addition to its canonical function to directly bind ETS sequences, *ETV6* seems to be recruited to additional sites, indicating that *ETV6* can act as a cofactor without direct DNA-binding. The most striking example is the high occurrence of RUNX motifs that could be explained by the *ETV6*-*AML1* fusion protein in Reh cells. However this is unique to the specific t(12;21)-positive background of these cells and might not reflect the general impact of *ETV6* in normal cells. Nonetheless, a significant proportion of peaks contained neither ETS nor RUNX motifs but were still associated to a change in gene expression. These peaks were enriched for IRF binding sites in combination or not with ETS sites. This result is supported by the known interaction between *ETV6* and *IRF8*^43^ and further suggests that the interaction between *ETV6* (and potentially other ETS factors) and IRF proteins is a rather common mechanism of transcriptional regulation. The function of *ETV6* as a cofactor is however poorly understood and additional efforts are required to uncover putative partners and characterize their functions.

All together, this report indicates that *ETV6* binding is highly flexible and therefore *ETV6* inactivation is expected to induce unique transcriptional modifications in a given cellular context. The wide spectrum of hematological diseases associated with *ETV6* alterations^5–10^ could be explained, at least partially, by the distinct *ETV6* regulatory network of the cell originally affected by the mutation. Accordingly, the complete disruption of *ETV6* observed in most t(12;21)-positive childhood pre-B ALL cases^17–21^ may induce specific transcriptional changes required for complete leukemic transformation. Although this study dissected *ETV6* binding and clarified its transcriptional network in t(12;21)-positive pre-B leukemia cells^32^, it remains challenging to associate *ETV6* target genes to leukemia-related phenotypes as their functions are, for most of them, still unclear.

## Conclusions

Molecular characterization of *ETV6* function is mandatory to fully understand its role in leukemogenesis. Towards this goal we built the first genome wide map of *ETV6* binding sites in pre-B leukemia cells bearing the recurrent t(12;21) translocation. By including expression data, we obtained the detailed transcriptional network of *ETV6* in these cells. This comprehensive analysis exposed the binding properties of *ETV6* and suggests that *ETV6* could also act as a cofactor to regulate gene expression. With the recent reports connecting germline *ETV6* mutations to a panel of familial hematological diseases and given the complexity of *ETV6*-mediated transcription, further characterization of *ETV6* remains valuable.

## Electronic supplementary material

Supplementary information

Table S3

Table S4

Table S6

Table S8

## Data Availability

The datasets generated and/or analysed during the current study are available in the GEO repository, https://www.ncbi.nlm.nih.gov/geo/query/acc.cgi?acc=GSE102785.
