# Supplementary material for: Genome wide mapping of ETV6 binding sites in pre-B leukemic cells
Source: Sci Rep. 2018 Oct 19;8:15526. doi: 10.1038/s41598-018-33947-1 (PMC6195514; doi:10.1038/s41598-018-33947-1)
Supplement: Supplementary file 1 — Supplementary information [file 41598_2018_33947_MOESM1_ESM.pdf]

Supplementary information

Genome wide mapping of *ETV6* binding sites in pre-B leukemic cells

Benjamin Neveu[1,2], Maxime Caron[1], Karine Lagacé[1,2], Chantal Richer[1] and Daniel Sinnett[1,2,3,*]

1) Sainte-Justine UHC Research Center, Montreal, Qc, Canada;

2) Department of Biochemistry and Molecular Medicine, Faculty of Medicine, University of Montreal, Montreal, Qc, Canada.

3) Department of Pediatrics, Faculty of Medicine, University of Montreal, Montreal, Qc, Canada.

*Corresponding author

Original Fig 1A

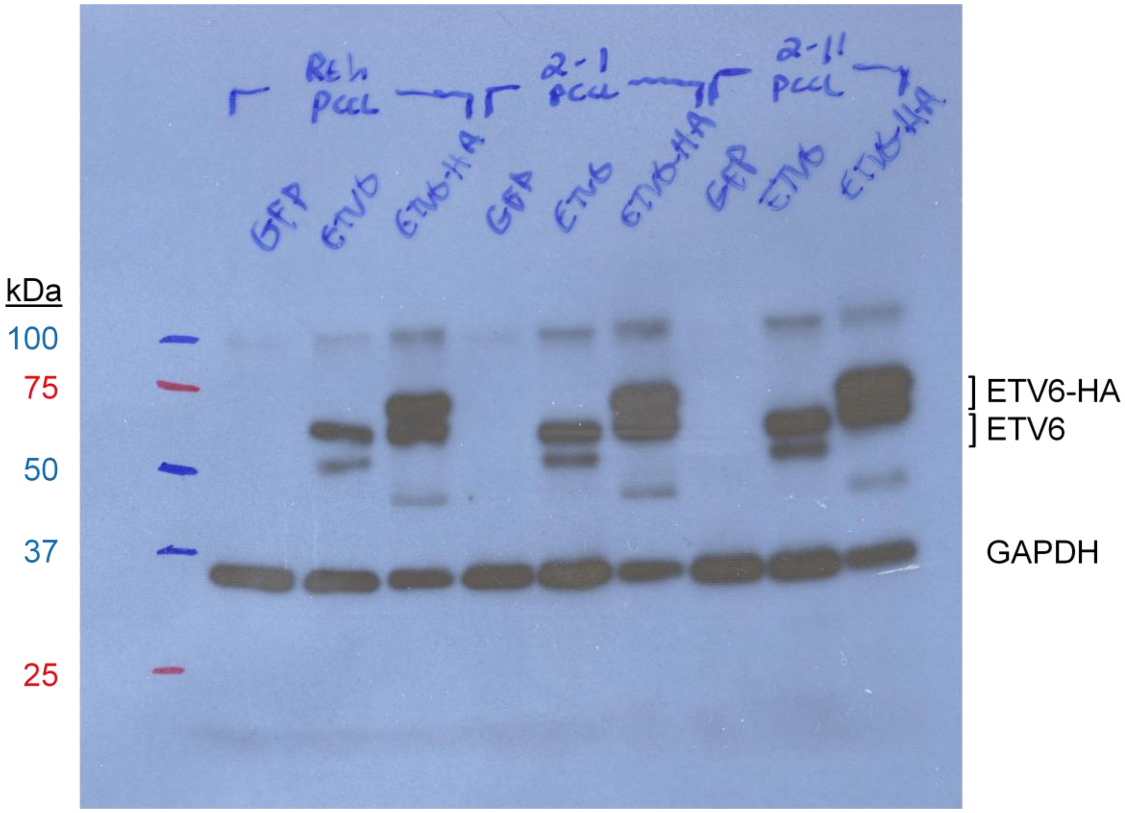

Supplementary figures

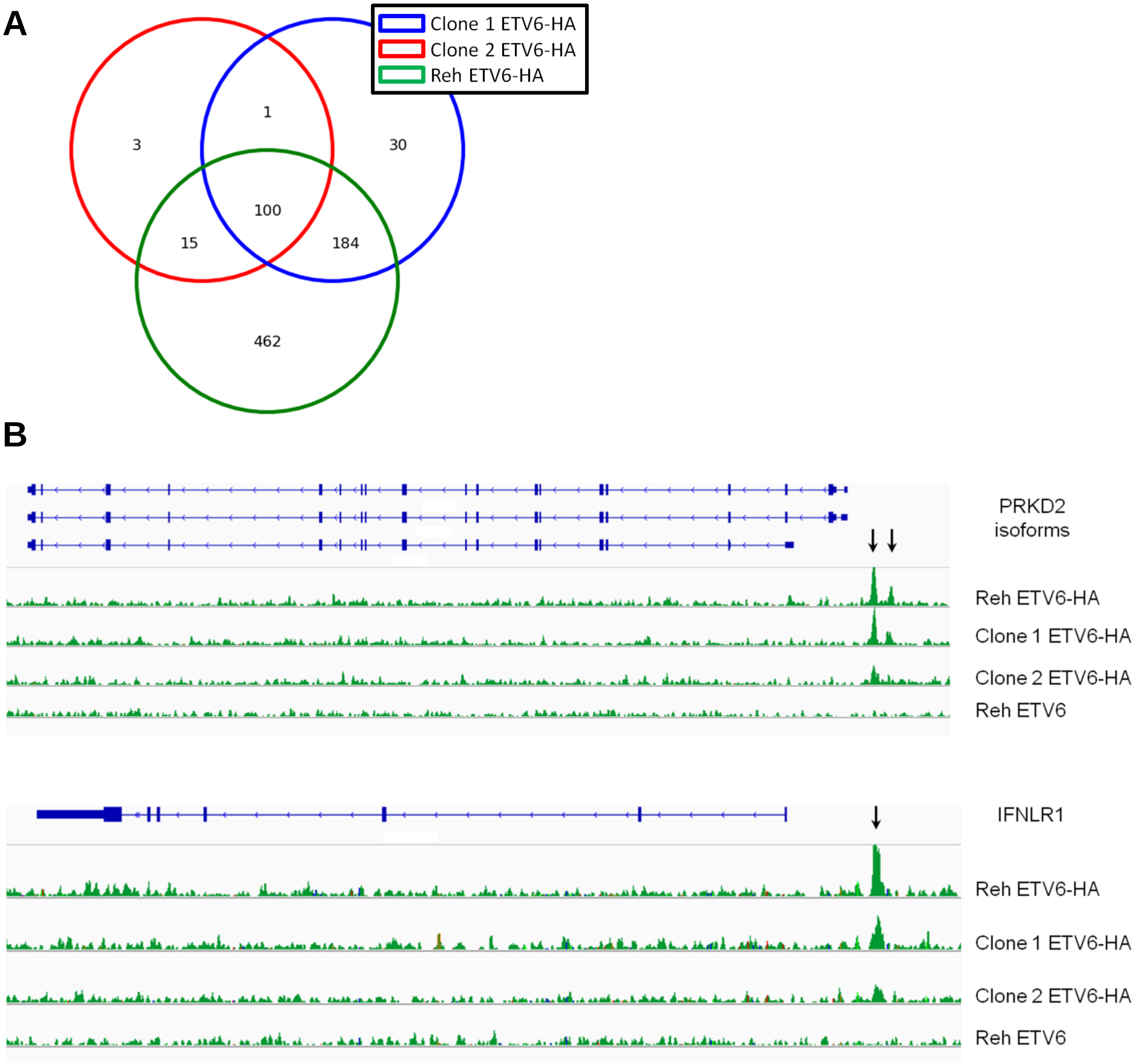

Fig S1: Comparison of the biological replicates. A. Overlap of the *ETV6* peaks identified in the 3 replicates. 90.2% and 96.6% of peaks called in Clone 1 and 2 were also identified in Reh cells, respectively. **B.** Examples of identified *ETV6* peaks. Distribution of the reads mapped to the *PRKD2* (upper panel) and *IFNLR1* (lower panel) genes after sequencing of the HA-immunoprecipitated DNA. Peaks in their promoter region (arrows) are seen in all three ETV6-HA populations but not in Reh ETV6 cells (negative immunoprecipitation control).

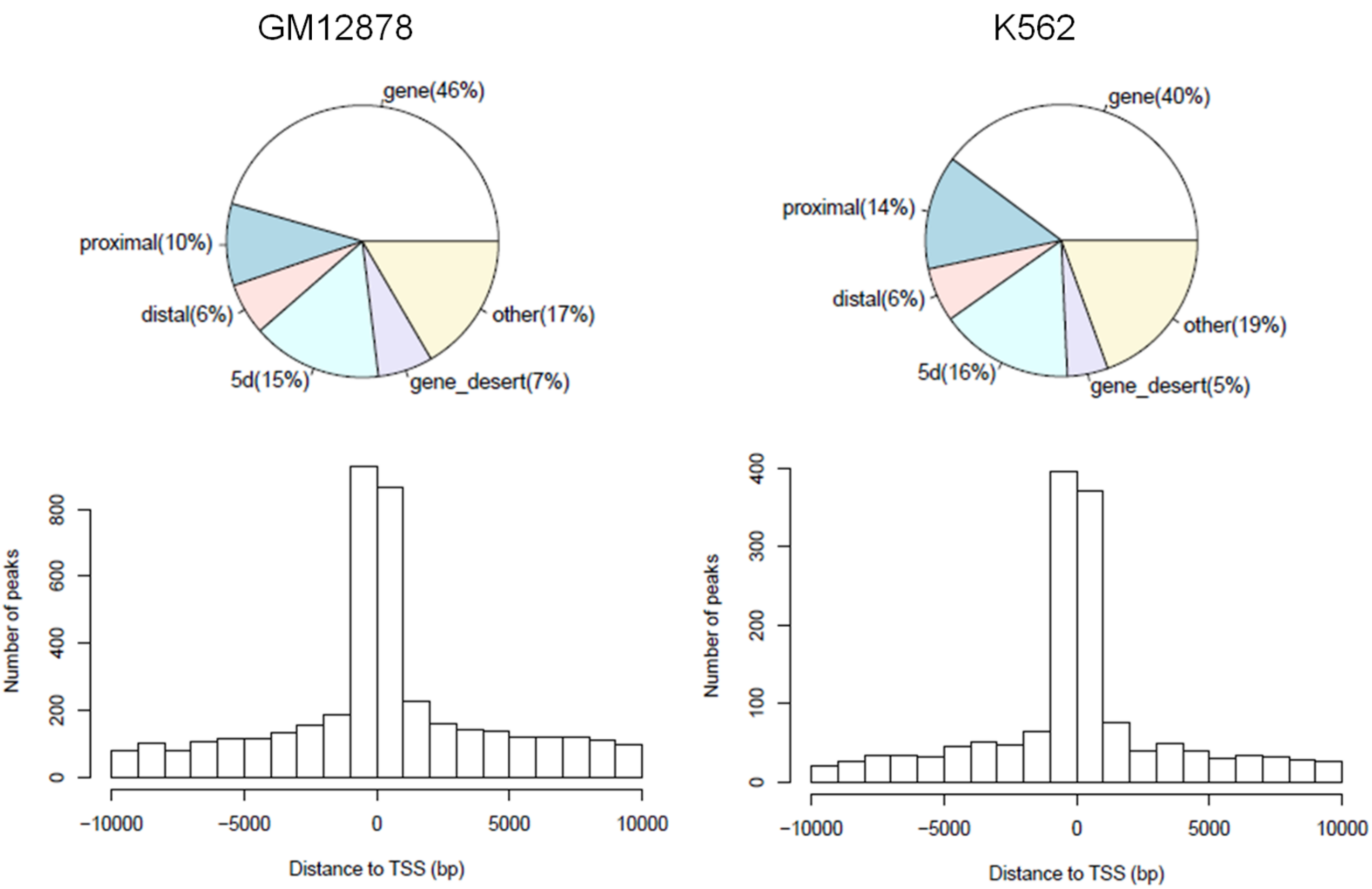

Fig S2: Genomic annotations of *ETV6* peaks in GM12878 and K562 cells. Genomic distribution of *ETV6* binding sites are shown in upper panels. Proximal:≤2kb upstream of TSS; Distal: 2kb to 10kb upstream of TSS; 5d: 10kb to 100kb upstream of TSS. Distribution of peaks in a 20 kb region spawning the TSS (+/- 10 kb) is shown in lower panels. *ETV6* peaks in both GM12878 and K562 cells display a clear TSS centered distribution.

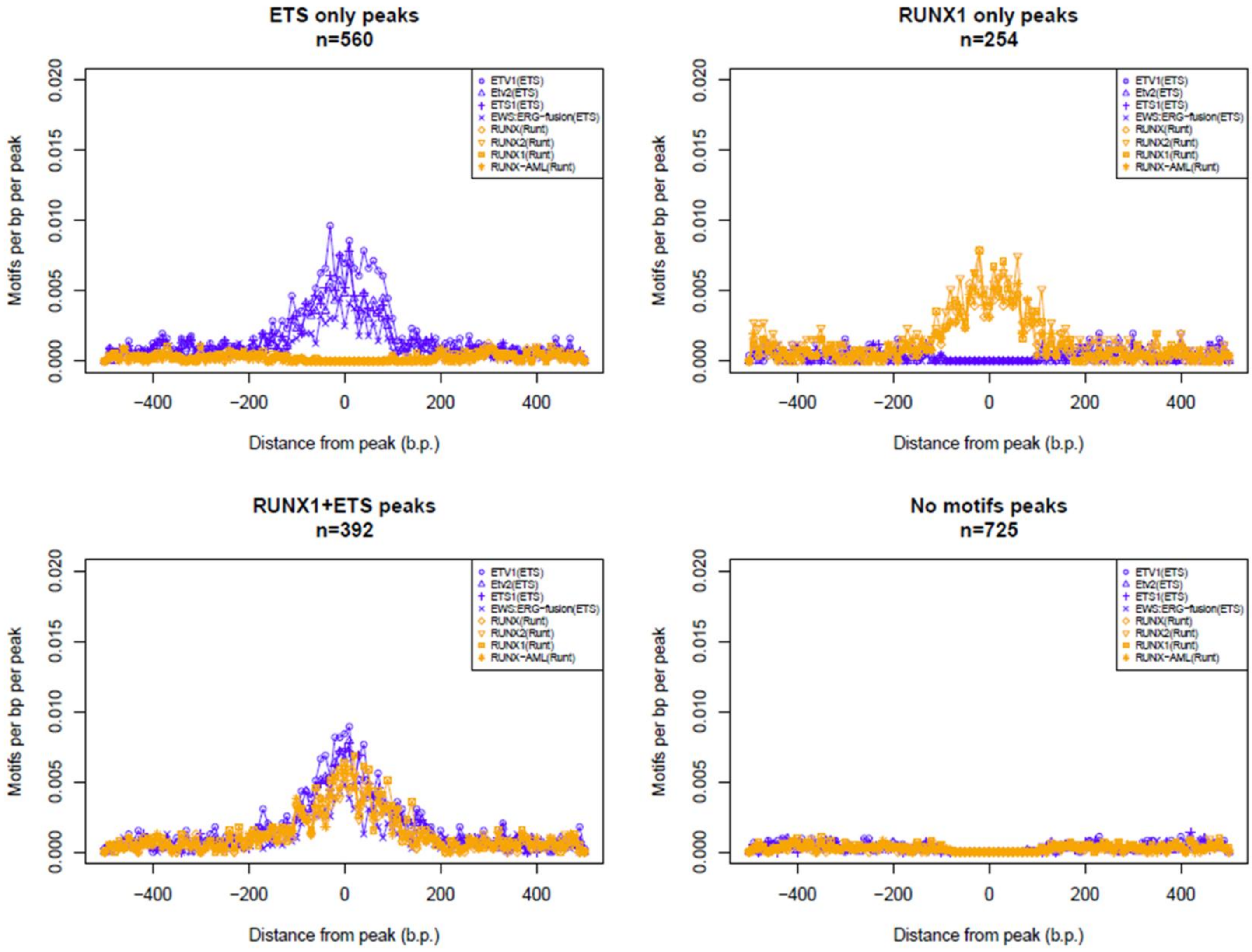

Fig S3: Density of the top 8 over-represented motifs in Reh *ETV6* peaks. The density of the 4 over-represented ETS motifs (blue) and RUNX motifs (yellow) identified in Reh-derived *ETV6*-bound regions is shown in a 1,000 bp window (+/- 500 bp) around the peak summits for each motif group. ETS and RUNX motifs are clustered to the peak summits.

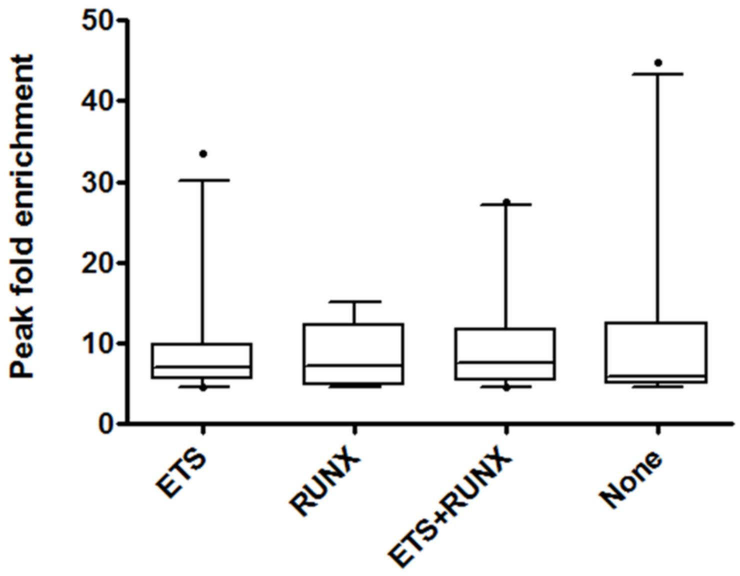

Fig S4: Fold enrichment of expression-correlated *ETV6* peaks in Reh cells according to each motif groups. Peak fold enrichments are not significantly different between motif groups among expression-correlated *ETV6* peaks (n=74). Whiskers represent the 5-95 percentiles. Statistical significance is calculated by two-tailed Student's *t* test.

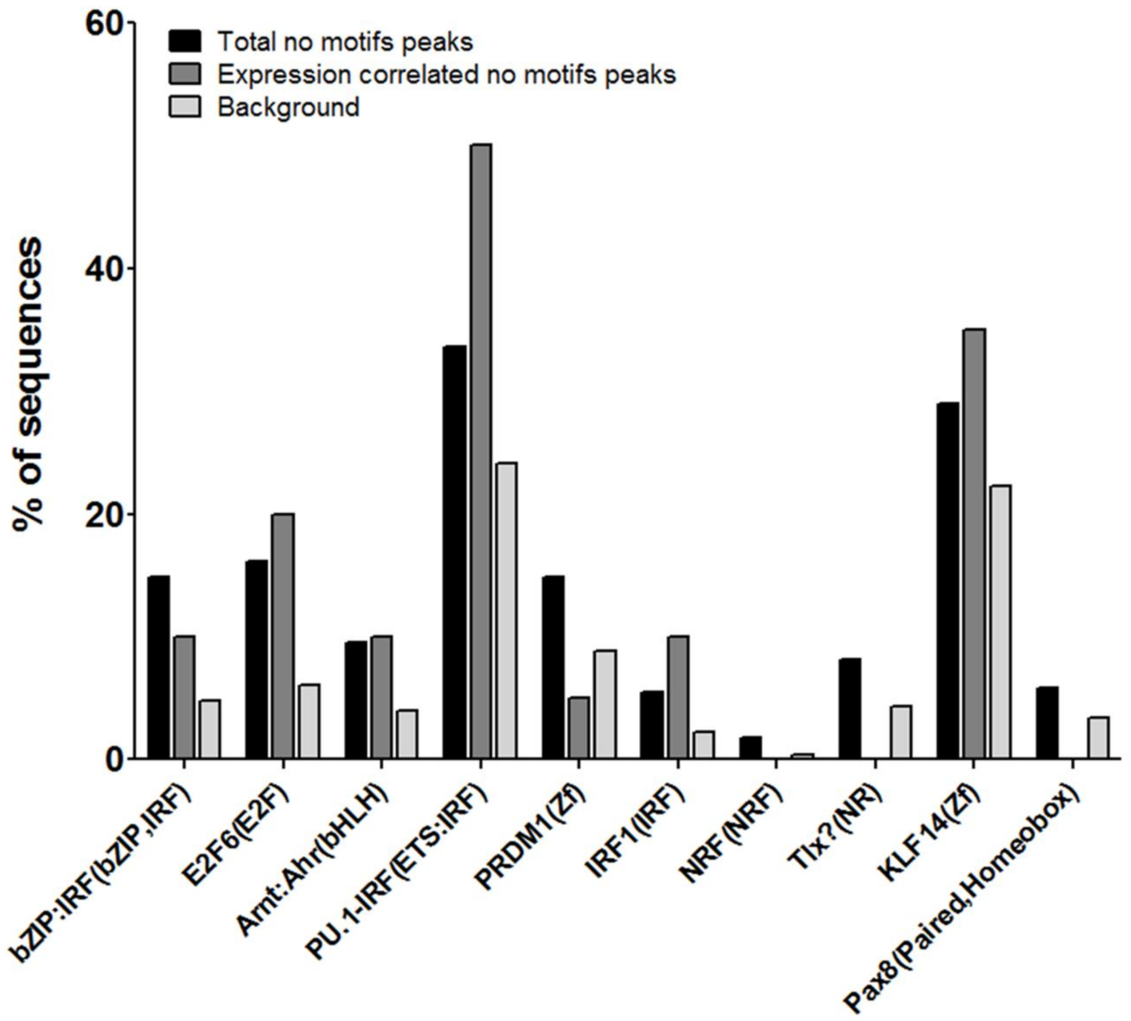

Fig S5: Motif enrichment analysis of ETS/RUNX free *ETV6* peaks in Reh cells. A second round of motif enrichment was performed on ETS/RUNX free *ETV6* peaks (n=725) which identified 10 significantly over-represented motifs (shown on *x* axis). The percentage of sequences containing each motif is given for the totality of ETS/RUNX free peaks (n=725), expression-correlated ETS/RUNX free peaks (n=20) and for the background (n= 33,996). Notably, an ETS-IRF compound motif (4th motif) was over-represented and strongly associated to expression regulation.

Supplementary tables

Larger tables (Supplementary Tables S3, S4, S6 and S8) are available online.

Table S1: Oligonucleotide sequences used for chromatin immunoprecipitation qPCR

Oligo Name	Sequence (5' to 3')
ChIP_CLIC5_A_F	CCAGCACCCTACTCTCCTTT
ChIP_CLIC5_A_R	CTCCACTACCAGCCTTACCC
Neg_F	ATGGTTGCCACTGGGGATCT
Neg_R	TGCCAAAGCCTAGGGGAAGA

Table S2: Summary statistics of Reh-derived *ETV6* peaks using different peak fold enrichment thresholds

Peak fold enrichment	Peaks	Associated genes	Total ensembl genes	Overlap RNA-seq (genes)	Total RNA-seq genes	Overlap ensembl (%)	Overlap RNA-seq (%)	Enrichment ratio	Fisher exact p-value
3	110 435	30133	60235	138	147	50	93.8	1.876	7,61E-31
3,1	60 248	24641	60235	124	147	40.9	84.3	2.061	3,19E-27
3,2	59 771	24584	60235	124	147	40.8	84.3	2.066	2,87E-27
3,3	31 801	18549	60235	113	147	30.7	76.8	2.501	2,09E-30
3,4	31 563	18495	60235	113	147	30.7	76.8	2.501	1,57E-30
3,5	17 036	12864	60235	97	147	21.3	65.9	3.093	4,75E-31
3,6	9 495	8438	60235	78	147	14	53	3.785	1,03E-28
3,7	9 399	8399	60235	78	147	13.9	53	3.812	7,55E-29
3,8	5 527	5478	60235	67	147	9	45.5	5.055	8,45E-31
3,9	5 475	5446	60235	66	147	9	44.8	4.977	4,95E-30
4	3 581	3792	60235	58	147	6.2	39.4	6.354	4,70E-31
4,1	3 549	3773	60235	58	147	6.2	39.4	6.354	3,62E-31
4,2	2 512	2778	60235	52	147	4.6	35.3	7.673	1,26E-31
4,3	2 489	2769	60235	52	147	4.5	35.3	7.844	1,08E-31
4,4	1 934	2223	60235	51	147	3.6	34.6	9.611	4,46E-35
**4,5**	**1 931**	**2223**	**60235**	**51**	**147**	**3.6**	**34.6**	**9.611**	**4,46E-35**
4,6	1 598	1891	60235	48	147	3.1	32.6	10.516	8,06E-35
4,7	1 595	1890	60235	48	147	3.1	32.6	10.516	7,87E-35
4,8	1 382	1675	60235	46	147	2.7	31.2	11.555	9,60E-35
4,9	1 376	1672	60235	46	147	2.7	31.2	11.555	8,89E-35
5	1 226	1516	60235	41	147	2.5	27.8	11.12	1,42E-30
5,1	1 223	1512	60235	41	147	2.5	27.8	11.12	1,28E-30
5,2	1 108	1391	60235	39	147	2.3	26.5	11.521	1,32E-29
5,3	1 009	1281	60235	38	147	2.1	25.8	12.285	1,09E-29
5,4	1 009	1281	60235	38	147	2.1	25.8	12.285	1,09E-29
5,5	923	1181	60235	37	147	1.9	25.1	13.21	1,04E-29
5,6	923	1181	60235	37	147	1.9	25.1	13.21	1,04E-29
5,7	846	1092	60235	36	147	1.8	24.4	13.555	1,24E-29
5,8	846	1092	60235	36	147	1.8	24.4	13.555	1,24E-29
5,9	790	1021	60235	36	147	1.6	24.4	15.25	1,31E-30
6	787	1019	60235	36	147	1.6	24.4	15.25	1,23E-30
6,1	736	956	60235	35	147	1.5	23.8	15.866	2,78E-30
6,2	736	956	60235	35	147	1.5	23.8	15.866	2,78E-30
6,3	683	897	60235	35	147	1.4	23.8	17	3,47E-31
6,4	681	895	60235	35	147	1.4	23.8	17	3,23E-31
6,5	641	843	60235	34	147	1.3	23.1	17.769	9,59E-31

6,6	641	843	60235	34	147	1.3	23.1	17.769	9,59E-31
6,7	603	800	60235	34	147	1.3	23.1	17.769	1,82E-31
6,8	603	800	60235	34	147	1.3	23.1	17.769	1,82E-31
6,9	568	762	60235	34	147	1.2	23.1	19.25	3,86E-32
7	531	715	60235	31	147	1.1	21	19.09	6,11E-29

Table S5: Reh and GM12878 overlapping peaks and correlated gene expression

	Reh *ETV6* ChIP-seq		Expression correlated	
	# peaks	# genes	# genes	gene overlap (%)
Total	1931	2223	51	2,29
GM overlap	386	457	14	3,06

Table S7: Peak fold enrichment per motifs and expression

		# peaks	Mean FC	p-value
**Motifs**	ETS	560	7.26	0.01710
	RUNX	254	6.58	0.00167
	ETS+RUNX	392	7.53	0.03823
	None	725	6.24	0.00035
**Expression correlated**		74	9.25	-

* p-values are calculated using peak fold changes (FC) in each motif group vs the "expression correlated" peaks
